# Robot-assisted resection of choledochal cyst in children

**DOI:** 10.3389/fped.2023.1162236

**Published:** 2023-06-19

**Authors:** Yi Jin, Shuhao Zhang, Duote Cai, Yuebin Zhang, Wenjuan Luo, Ken Chen, Qingjiang Chen, Zhigang Gao

**Affiliations:** Department of General Surgery, Children’s Hospital, Zhejiang University School of Medicine, National Clinical Research Center for Child Health, Hangzhou, China

**Keywords:** robot-assisted, choledochal cyst, resection, surgery, children

## Abstract

**Background:**

The emergence of the robotic surgery system has assisted the further development of minimally invasive surgery by facilitating more delicate and precise complex procedures. The purpose of this study was to present a study of robot-assisted resection of the choledochal cyst and to discuss the technical points.

**Methods:**

In total, 133 patients who were diagnosed with a choledochal cyst and underwent surgery from April 2020 to February 2022 in the Children's Hospital, Zhejiang University School of Medicine, were retrospectively analyzed. The data were collected including the clinical information of the patients, operative details, and postoperative outcomes.

**Results:**

Among these 133 patients, 99 underwent robot-assisted surgery and 34 underwent laparoscopic assisted surgery. The median operation time was 180 min, with an interquartile range (IQR) of 170–210 min for the robot-assisted group and 180 min with an IQR of 157.5–220 min in the laparoscopic assisted group (*P* = 0.290). The detection rate of 82.5% for the distal opening of the cystic type of choledochal cyst was higher in the robot-assisted group than that in the laparoscopic assisted group at 34.8% (*P* = 0.000). The postoperative hospital stay was shorter (*P* = 0.009) and the hospitalization expense was higher (*P* = 0.000) in the robot-assisted group than that of the laparoscopic assisted group. There was no significant difference between the two groups in terms of complications, postoperative indwelling days of the abdominal drainage tube, intraoperative blood loss, and postoperative fasting time (*P* > 0.05).

**Conclusions:**

Robot-assisted resection of choledochal cyst is safe and feasible, it is ideal for the patient requiring a meticulous operation, and its postoperative recovery was shorter than for traditional laparoscopy.

## 1. Introduction

The choledochal cyst (CC) is a rare congenital dilation of the bile duct, which is much more common in Asian countries (1); since the first report of laparoscopy to excise a choledochal cyst in 1995 (2), its wide spread use has effectively replaced traditional open surgery (3). Compared with the conventional laparoscopic surgery, robotic surgery enhances several key aspects of minimally invasive surgery, the instrument has several advantages such as three-dimensional magnification imaging, jitter filtering function, multiple degree of freedom rotation of the robotic arm, and high comfort of the surgeon operation (4), Woo et al. first reported the robot-assisted resection of choledochal cyst in children in 2006 (5), and subsequent reports have noted the feasibility and safety of the robot-assisted surgery (6, 7).

The present study aimed to present the author's experience in robot-assisted excision of choledochal cysts, discuss technical issues, and compare the surgical results of laparoscopic and robot-assisted removal.

## 2. Materials and methods

### 2.1. Clinical information

A retrospective study was conducted on 133 patients with choledochal cyst who underwent surgical resection from April 2020 to February 2022 at the Children's Hospital, Zhejiang University School of Medicine, and the design was approved by the institute’s ethics committee (No. 2020-IRB-055). Whether the patient underwent conventional laparoscopic surgery or robot-assisted surgery depended on the surgeon and the patient's parents’ preference. Patients with a history of abdominal surgery were excluded.

### 2.2. Surgical procedures

#### 2.2.1. Robot-assisted surgery

The Da Vinci Xi Surgical System was used to perform the surgery.

Step one: port arrangement.

**Figure 1:**
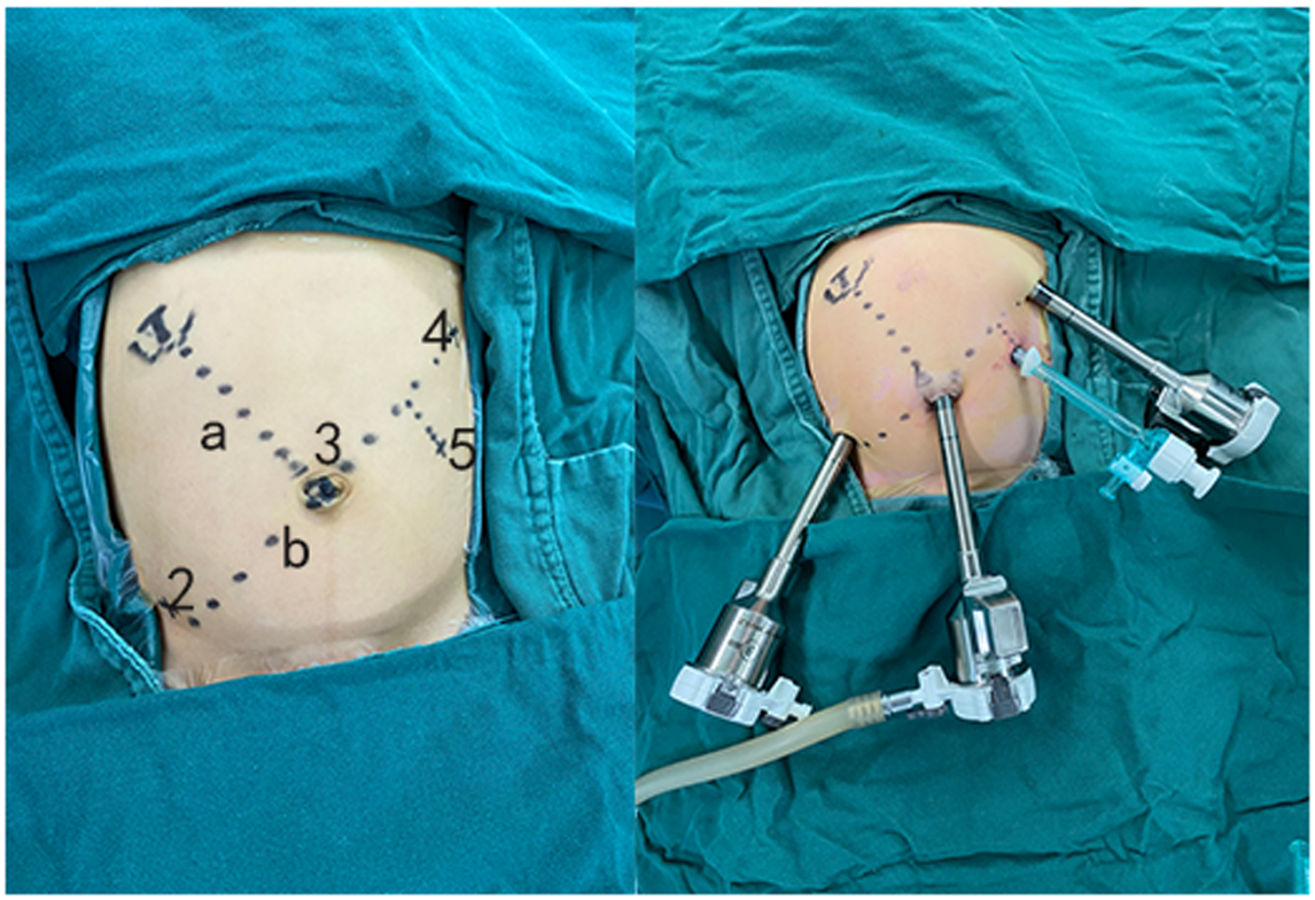
Take the umbilicus and the right upper abdomen (hepatic hilum body surface projection) as two points to make a line “a,” and make a line “b” perpendicular to line “a” through the umbilicus (No. 3). Take two points (No. 2 and No. 4) on the line b about 8 cm away from the umbilicus (at least 3 cm), No. 2–4 are the positions of the Da Vinci Trocar (8 mm), and No. 5 is the laparoscopic port (5 mm) for the assistant surgeon.

The patient was placed in the supine position and after pneumoperitoneum was established, an 8 mm Da Vinci trocar was inserted at the umbilical incision, which was used as a port for the 3D camera. Another two 8 mm trocars were placed at the left upper abdomen and right lower abdomen about 8 cm (at least 3 cm) from the umbilicus incision, and a 5 mm laparoscopic trocar was placed at the left abdomen as an auxiliary port for the for the assistant surgeon (Figure 1).

Step two: extracorporeal jejunojejunostomy.

The proximal jejunum 15–20 cm from the ligament of Treitz was identified and captured with a laparoscopic grasper under endoscopic monitoring, then the umbilical incision was extended, and the marked jejunum was exteriorized. After the jejunal Roux limb was fashioned extracorporeally, the middle transverse colon was exteriorized and the biliary loop was placed to retrocolon through the right mesentery of the transverse colon. Then, all intestines were sent back to the abdominal cavity, and the umbilical incision was partially closed to reinstall the Da Vinci trocar.

Step three: docking.

We then change the patient into a Trendelenburg position, then re-establishing the pneumoperitoneum, after setting the machine to “epigastrium surgery” mode, no. 1 arm was vacant, no. 3 arm was connected to the umbilical trocar, entering the main mirror to confirm the surgical field, then press the “targeting” button to adjust other mechanical arms, then no. 2 and 4 robotic arms were connected to the trocar at points 2 and 4, respectively (Figure 1), and the operating instruments were installed under the monitoring of the main mirror. The no. 2 trocar was placed with a dissector, and no. 4 trocar was placed with a needle holder or electric hook.

Step four: robot surgery.

**Figure 2:**
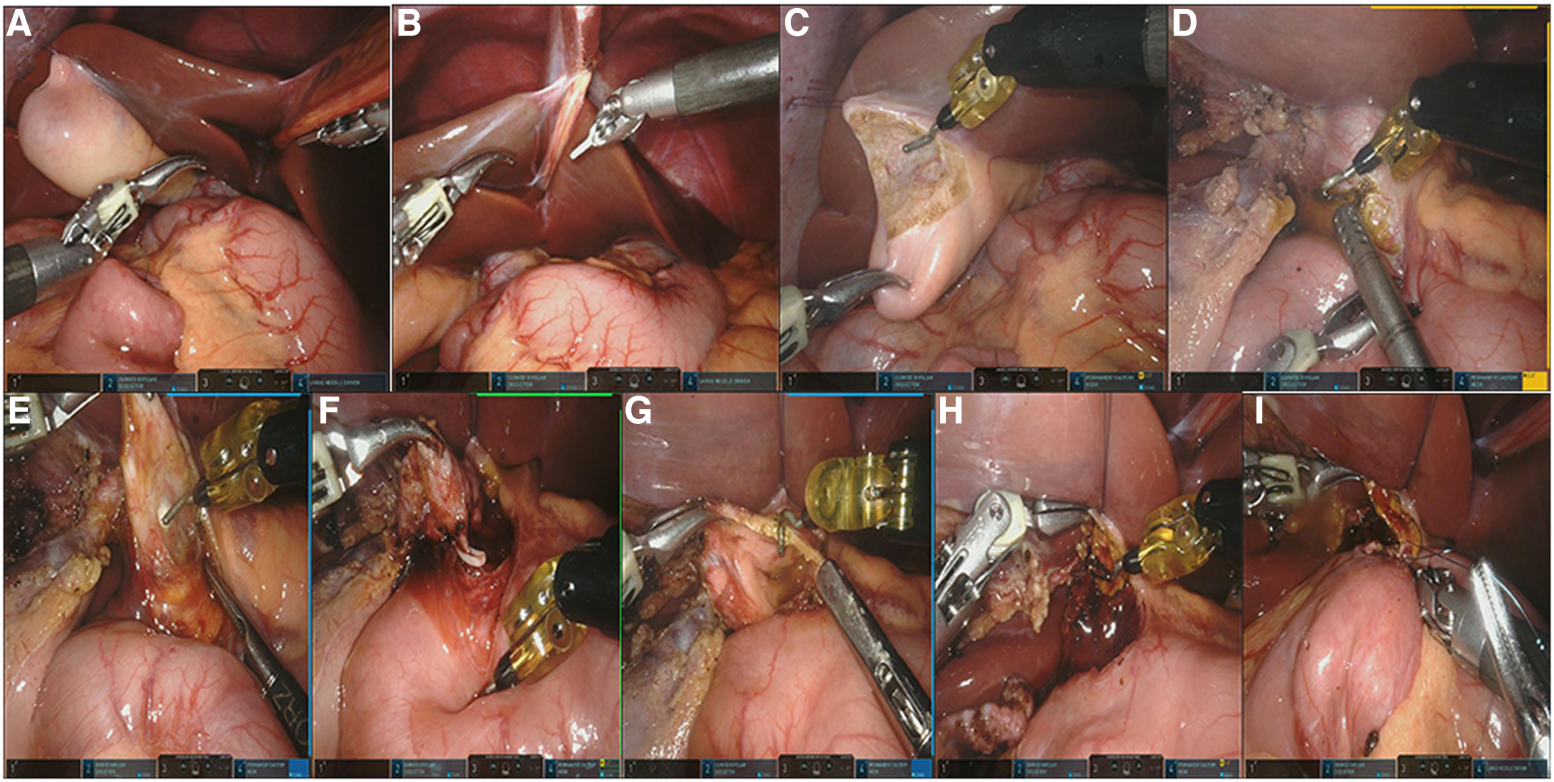
(**A,B**) Suspending the ligamentum teres hepatis and gallbladder fossa; (**C**) removing the gallbladder with electric hook; (**D**) decompression the cyst; (**E**) dissecting the cyst; (**F**) ligating the distal opening of the choledochal cyst with Hem-o-Lok polymeric clips; (**G,H**) detaching the proximal end of the cyst and trimming the hepatic duct of the hilar part; (**I**) the end-to-side hepaticojejunostomy.

Two surgeons perform the robotic part, including one chief surgeon and one assistant. To expose the hilar, we suspended the gallbladder fossa and ligament teres by using a 3-0 absorbable sliding line (Figures 2A,B). The gallbladder was the first target of resection (Figure 2C). We then incise the anterior wall of the cyst, followed by decompression and washing the stones or protein plugs, then peeling off the cyst gradually (Figures 2D,E). The distal end of the cyst was dissected as close as possible to the common channel and ligated using Hem-o-Lok polymeric clips (Figure 2F). There is currently no consensus on how long the distal end of the cyst should be removed; based on our experience, we measure the distance between the cyst and the common duct through preoperative Magnetic resonance cholagiopancreatography (MRCP), and roughly peel the distal end of the cyst to this length during surgery; for cases in whom preoperative MRCP cannot clearly display the common duct, we will peel the distal end to a diameter of approximately 1 mm and then clamp it with Hem-o-Lok and the distal end, which has no obvious opening and is directly disconnected. The proximal end of the cyst is detached (Figures 2G,H), and then 4-0 absorbable sutures for hepaticojejunostomy is applied (Figure 2I). After operation, the drainage tube was placed in the hepatorenal crypt under endoscopic monitoring, the pneumoperitoneum was then decompressed, specimen was removed through umbilical incision, and the incision was sutured closed.

#### 2.2.2. Laparoscopic assisted surgery

Four ports were used to perform the surgery. A 3-0 sliding line was used to suspend the ligament teres and gallbladder fossa to expose the hilar and cyst for surgical convenience. Under laparoscopy, the anatomy of the biliary tract and the removal of choledochal cyst and gallbladder were completed. We then incise the anterior wall of the cyst, followed by decompression and washing, then peeling off the cyst gradually. The distal end of the cyst was dissected as close as possible to the common channel and ligated with Hem-o-Lok polymeric clips or sutured with 5-0 sliding line; the distal end that had no obvious opening was directly disconnected and then the proximal end of the cyst to the hepatic duct of the hilar part was separated. The proximal jejunum 15–20 cm from the ligament of Treitz was identified and captured with a laparoscopic grasper under endoscopic monitoring, and then exteriorized through the extended umbilical incision. The jejunal Roux limb was fashioned extracorporeally, and then the middle transverse colon was exteriorized; the biliary loop was placed in the retrocolon through the right mesentery of the transverse colon. All intestines were then returned to the abdomen and the umbilical wound was partially closed to refit the 5-mm trocar; the pneumoperitoneum was re-established and a 4-0 absorbable suture was used for end-to-side hepaticojejunostomy. After the operation, a drainage tube was placed around the anastomotic site under endoscopic monitoring, and the choledochal cyst and gallbladder were removed through the umbilical incision.

#### 2.3. Statistical analysis

Statistical analysis was performed using SPSS 24.0 software. Continuous variables were reported as median and interquartile range (IQR), and Mann–Whitney *U* test was used to compare data from the two groups. Enumeration data were expressed as rate (%), and the comparison between the two groups used Pearson's *χ*^2^ and Fisher's exact tests. *P* < 0.05 was considered statistically significant.

## 3. Results

Ninety-nine patients (25 males and 74 females) received robot-assisted surgery and 34 patients (4 males and 30 females) received conventional laparoscopic assisted surgery. Two cases in the Da Vinci surgery group were found to have accessory hepatic ducts during surgery, while no cases of biliary tract variation were found in the laparoscopic surgery group. There were five cases in the Da Vinci surgery group and two cases in the laparoscopic surgery group with a diameter of hepaticojejunostomy less than 5 mm, respectively. There was no significant difference in patients’ age, weight, operation time, and the postoperative feeding time between two groups (*P* > 0.05). The robot-assisted group had a significantly higher hospitalization cost (*P* < 0.05) and shorter postoperative hospitalization time, compared with the laparoscopic assisted group as seen in Table 1.

**Table 1:** Clinical parameters of the patients.

Group	*n*	Gender (male/female)	Age [months, M (IQR)]	Weight [kg, M (IQR)]	Surgical time [min, M (IQR)]	Intraoperative bleeding [ml, M (IQR)]	Postoperative feeding time [days, M (IQR)]	Postoperative hospital stay [days, M (IQR)]	Abdominal drainage tube [days, M (IQR)]	Hospitalization expense [RMB, M (IQR)]	Complications (*n*)
Robot-assisted surgery	99	25/74	34 (13–56)	13.5 (9.5–16.2)	180 (170–210)	5 (5–10)	4 (4–5)	9 (8–12)	7 (6–9)	80,382 (74,457–87,171)	5
Laparoscopic assisted surgery	34	4/30	24 (2.875–44.5)	11.25 (5.975–14.7)	180 (157.5–220)	7.5 (5–12.5)	5 (4–5)	10.5 (9–13)	7 (7–8)	33,822.5 (28,852–40,679)	3
*Χ*^2^/*t*/Z	—	2.7	−1.79	−2.18	−1.059	−1.432	−0.275	−2.642	−0.991	−8.294	0.637
*P*	—	0.1	0.073	0.029	0.290	0.152	0.783	0.009	0.322	0.000	0.425

M, mean; IQE, interquartile range.

The surgical time in robot-assisted surgery included installation time (from skin to skin).

There were 63 cases of cystic-type choledochal cyst in the robot-assisted group, and in 52 (82.5%) cases, the distal end could be clearly exposed and dissected for ligation. In 23 cases of cystic-type choledochal cyst in the laparoscopic assisted group, 8 cases (34.8%) could have the distal end exposed and dissected. For other cases, the cyst was directly peeled off without detecting the distal end.

One case in the robot-assisted group and two cases in the laparoscopic assisted group were converted to laparotomy, and there was no significant difference in the conversion rate of laparotomy (*P* = 0.161). For the robot-assisted group, this case was a 10-year-old boy with a 13 cm × 9.4 cm cyst, the cyst wall was hypertrophic and edematous, and the blood vessels were thick and proliferative. During the anatomy of the cyst, there was rapid bleeding on the peeling surface of the cyst wall, and it was difficult to stop the bleeding under the endoscope, so it was converted to laparotomy. For the laparoscopic assisted group, one case was a 5-year-old boy with a 10 cm × 7.9 cm cyst; extensive necrosis was found on the posterior wall of the cyst during operation, which led to an unclear tissue gap, and the portal vein was injured during the anatomy of the cyst. The other case was a 7-month-old girl with a 2 cm × 2 cm cyst, with a particularly small common hepatic duct of 1 mm diameter, where it was necessary to dissect the hepatic hilum, which is difficult to operate under endoscopy.

In the robot-assisted group, postoperative complications included one postoperative incisional hernia, one postoperative hemorrhage, and three postoperative bile fistulae. In the laparoscopic assisted group, these included one postoperative bile fistula and one postoperative pancreatic fistula. Except for the incision hernia patient in the robot-assisted group and the biliary fistula patients in the laparoscopic group, which were cured by secondary operation, the other patients were healed by conservative treatment. For cases of postoperative bile fistula, due to the patient's stable vital signs and no obvious signs of peritonitis, we first adopted conservative treatment including abrosia, antibiotic anti-infection, total parenteral nutrition, and somatostatin inhibition of glandular secretion. Among them, three patients in the Da Vinci surgical group were cured after conservative treatment (the drainage volume of the abdominal drainage tube gradually decreased) and did not undergo further surgery, one patient in the laparoscopic surgery group continued to have bile like drainage fluid that could not be reduced after conservative treatment, and gradually developed fever and white stools, leading to the second laparotomy to repair the biliary fistula. Six months of follow-up after surgery, all patients recovered very well, and the abdominal incision was well healed with no significant difference between the two groups.

## 4. Discussion

The Da Vinci robotic surgical system has set off a boom in the field of adult surgery because of its series of designs that are conducive to precise operation during surgery (8). However, this system requires multiple large caliber trocar incisions and the cost is expensive; thus, the application in the field of pediatric surgery is relatively small. With the accumulation of cases of Da Vinci robot-assisted choledochal cyst surgery and the increase of relevant clinical reports, this operation is becoming more popular and its feasibility is being recognized (9, 10).

According to our experience, Da Vinci robotic surgery has the following advantages compared to the laparoscopic surgery: (1) less side injury during anatomy; (2) more reliable treatment of the distal end of the cyst; (3) greater suitability for a hepaticojejunostomy with a diameter less than 5 mm; and (4) faster postoperative recovery and shorter length of hospitalization.

Dissociation and ligation of the distal end of the cyst are the routine procedures of choledochal cyst resection, but most of the distal openings of cystic choledochal cysts are small (11), which makes conventional laparoscopy difficult. Some children will develop pancreatitis after resection of the choledochal cyst, which may be caused by directly disconnecting the distal opening (12) and a remnant of the distal cyst may lead to the development of cholangiocarcinoma in the future (13). Overall, the Da Vinci surgical system makes it easier to detect and free the distal opening and ligate it.

The hepaticojejunostomy with a diameter less than 5 mm is a test of the surgeon's surgical skills in laparoscopic surgery, due to unclear vision and unsatisfactory suture angle, resulting in an increased risk of postoperative anastomotic leakage or stenosis. The Da Vinci surgical system has greater magnification and a precise multiangle rotating manipulator, which improves the success rate of treatment of anastomotic stoma with a diameter less than 5 mm.

Anecdotal evidence is that postoperative discomfort such as abdominal pain and wound pain for children in the Da Vinci operation group was less than the laparoscopic operation group, which may be related to the fact that the Da Vinci trocar has no obvious traction on the abdominal wall under the control of mechanical arm and patients are more willing to accept early out of bed activities.

The Da Vinci system has shortcomings in the high total hospitalization costs and long installation time. A major barrier is the high surgical cost of the Da Vinci system, which is at the patient's own expense (14) and may resolve with time. The problem of time-consuming installation has been solved by the replacement of the Da Vinci Si system with the Da Vinci Xi system, which significantly shortened the installation time (15), and with the rapid development of artificial intelligence and mechanical engineering, the problem of docking time will be solved in the future.

The limitations of the present study are its retrospective nature and small sample size; in order to validate the advantages of this technique, a prospective larger, multicenter clinical trial and long-term follow-up is necessary.

## 5. Conclusions

Robot-assisted resection of choledochal cyst is feasible and safe, and compared with laparoscopic choledochal cyst resection, it is more suitable for cases that require precise procedures; its postoperative recovery speed is also faster than that of laparoscopic surgery. However, at present, some problems such as the high hospitalization cost and the lack of established indications for young children are still obstacles that need to be overcome.

## Data Availability

The original contributions presented in the study are included in the article, further inquiries can be directed to the corresponding author.
